# Mechanistic Target of Rapamycin Complex 1/S6 Kinase 1 Signals Influence T Cell Activation Independently of Ribosomal Protein S6 Phosphorylation

**DOI:** 10.4049/jimmunol.1501473

**Published:** 2015-10-09

**Authors:** Robert J. Salmond, Rebecca J. Brownlie, Oded Meyuhas, Rose Zamoyska

**Affiliations:** * Institute of Immunology and Infection Research, Ashworth Laboratories, University of Edinburgh, Edinburgh EH9 3FL, United Kingdom; and; † Department of Biochemistry and Molecular Biology, Institute for Medical Research Israel-Canada, The Hebrew University Hadassah Medical School, 91120 Jerusalem, Israel

## Abstract

Ag-dependent activation of naive T cells induces dramatic changes in cellular metabolism that are essential for cell growth, division, and differentiation. In recent years, the serine/threonine kinase mechanistic target of rapamycin (mTOR) has emerged as a key integrator of signaling pathways that regulate these metabolic processes. However, the role of specific downstream effectors of mTOR function in T cells is poorly understood. Ribosomal protein S6 (rpS6) is an essential component of the ribosome and is inducibly phosphorylated following mTOR activation in eukaryotic cells. In the current work, we addressed the role of phosphorylation of rpS6 as an effector of mTOR function in T cell development, growth, proliferation, and differentiation using knockin and TCR transgenic mice. Surprisingly, we demonstrate that rpS6 phosphorylation is not required for any of these processes either in vitro or in vivo. Indeed, rpS6 knockin mice are completely sensitive to the inhibitory effects of rapamycin and an S6 kinase 1 (S6K1)–specific inhibitor on T cell activation and proliferation. These results place the mTOR complex 1-S6K1 axis as a crucial determinant of T cell activation independently of its ability to regulate rpS6 phosphorylation.

## Introduction

Naive T cells undergo a rapid switch from quiescence to a highly metabolically active state upon recognition of cognate Ag. In recent years, it has become apparent that this metabolic reprogramming is critical not only for T cell growth and population expansion but also effector-memory differentiation during immune responses (1). Consequently, much research has focused on delineating the signaling pathways that regulate these metabolic changes and has identified the mechanistic target of rapamycin (mTOR) as a central player in T cell fate decisions.

mTOR is an evolutionarily conserved serine/threonine kinase that is expressed in cells as a component of two distinct functional complexes (reviewed in Refs. 2–5). Thus, mTOR complex 1 (mTORC1), composed of mTOR, raptor and mammalian lethal with SEC13 protein 8 (mLST8), is acutely sensitive to the immunosuppressive macrolide rapamycin. By contrast, the activity of mTORC2, consisting of mTOR, rictor, mammalian stress-activated protein kinase interacting protein 1, and G protein β subunit-like, is reduced only upon prolonged exposure to rapamycin. Although the suppressive and modulatory effects of rapamycin on immune responses have long been established, genetic evidence for an important role for mTOR in T cells has been provided by studies of T cell–specific deletion of mTOR (6), mTOR interacting proteins (7–10) and modulators of mTOR activity (11, 12). Taken together, these studies indicate that mTORC1 and mTORC2 have distinct roles in the regulation of CD4^+^ Th cell differentiation (7–9). Genetic ablation of mTOR itself, abrogating both mTORC1 and mTORC2 function, prevents the development of Th1, Th2, and Th17 responses and instead favors differentiation of regulatory T cells, irrespective of the polarizing cytokine milieu (6). Furthermore, in CD8^+^ T cells, the magnitude of mTOR signaling determines effector-memory differentiation. Thus, inhibition of mTOR activity by rapamycin treatment impairs the metabolic changes required for CD8^+^ effector cell differentiation and instead favors the generation of memory T cells in vivo (13–15).

Despite recent advances in our understanding of the roles of mTOR in T cell activation, the downstream signaling pathways and mechanisms by which mTOR exerts its effects remain somewhat obscure. Downstream of mTORC2, the serine/threonine kinase serum and glucocorticoid regulated kinase 1 regulate Th2 differentiation by preventing degradation of the JunB transcription factor and repressing IFN-γ production (16). The canonical targets of mTORC1 are the p70 ribosomal protein S6 kinase 1 (S6K1) and initiation factor 4E-binding proteins (4E-BPs). S6K1 is a key regulator of cellular metabolism and S6K1-deficient mice are smaller than wild-type littermates and display hypoinsulinemia and glucose intolerance (17). To mediate its effects on metabolic pathways, S6K1 phosphorylates a number of downstream substrates including the small ribosomal subunit protein S6 (rpS6). In T cells, rpS6 is phosphorylated on five evolutionarily conserved serine residues by S6K1 and to a lesser extent by other AGC kinases including the p90 ribosomal S6 kinases (18) in response to TCR/costimulation and cytokine and nutrient signaling pathways. rpS6 is critical for ribosome biogenesis and consequently germline deletion of *Rps6* is embryonically lethal (19) whereas T cell–specific deletion using CD4-Cre completely abrogates thymic T cell development (20). By contrast, the role of rpS6 phosphorylation is less well understood. Knockin mice in which all five phosphorylatable serine residues are substituted for alanine (rpS6^P−/−^) are viable (21), and rpS6^P−/−^ knockin mice recapitulate some but not all of the metabolic defects reported for S6K1-deficient animals (21, 22), indicating that in some cell types rpS6 phosphorylation is a key downstream effector of S6K1.

In T cells, activation of S6K1 and entry into the cell cycle and proliferation have long been linked (23–25); however, direct evidence of the precise roles for S6K1 and its downstream effectors in T cell responses is lacking. In the current work, using rpS6^P−/−^ knockin mice, we investigated the role of rpS6 phosphorylation as a downstream effector of mTORC1/S6K1 in T cell development, activation, and differentiation. Surprisingly, our data suggest that rpS6 phosphorylation is dispensable for T cell immune responses. Importantly, wild-type (WT) and rpS6^P−/−^ T cells are equally sensitive to the inhibitory effects of rapamycin and S6K1-specific inhibitors, indicating a vital role for mTORC1/S6K1 in T cell activation and differentiation independent of rpS6 phosphorylation.

## Materials and Methods

### Mice and *Listeria monocytogenes* infection

rpS6^P−/−^ mice (21) were backcrossed to the C57BL/6J genetic background more than eight times; backcrossed mice were further crossed to a *Rag1^−/−^* OT-I background (26). For infection experiments, groups of mice were inoculated i.v. with 10^6^ CFU of an attenuated (ActA mutant) ova-expressing strain of *L. monocytogenes* (27) (Lm-Ova; a gift from H. Shen, University of Pennsylvania, Philadelphia, PA). Mice were maintained and procedures performed in accordance with U.K. Home Office regulations at the University of Edinburgh.

### Cell culture and stimulation

Lymph node (LN) OT-I T cells were cultured in RPMI 1640 medium (Invitrogen) supplemented with 10% FCS, l-glutamine, antibiotics, and 50 μM 2-ME. SIINFEKL (N4), SIITFEKL (T4) and SIIGFEKL (G4) peptides (Peptide Synthesis) were added to culture media at the concentrations stated in figure legends. In some experiments, cells were cultured in the presence of 100 nM rapamycin or 10 μM S6K1 inhibitor PF-4708671 (both Tocris Bioscience). These conditions have previously been optimized for the selective inhibition of target kinases by the drugs (28–30). For CTL generation, OT-I T cells were stimulated with 10 nM N4 for 2 d, washed, and then differentiated in the presence of either recombinant human IL-2 or mouse IL-15 (both PeproTech) for an additional 4d. For cytokine recall responses, in vitro–generated CTLs or ex vivo polyclonal splenic T cells from Lm-Ova–infected mice were restimulated with peptide for 4 h in the presence of 2.5 μg/ml brefeldin A (Sigma-Aldrich).

### Flow cytometry and Abs

Fluorescently conjugated Abs were purchased from eBioscience, BD Pharmingen, and BioLegend. For intracellular staining of phospho-rpS6 S235/6 and S240/4 and phospho-ERK T202/Y204, cells were permeabilized using Phosflow Fixation/permeabilization buffer (BD Biosciences) and stained with phospho-specific rabbit mAb (Cell Signaling Technology) and anti-rabbit secondary Abs. Ova-specific T cells from Lm-Ova infection experiments were identified using PE-conjugated SIINFEKL-H-2K^b^ dextramers (Immudex). Data were acquired using a MacsQuant flow cytometer (Miltenyi Biotec) and analyzed using FlowJo software (Tree Star).

### Statistical analysis

Two-tailed paired or unpaired Student *t* tests were performed using Prism software. The Holm–Sidak correction for multiple comparisons was used where appropriate. The *p* values < 0.05 were considered statistically significant.

## Results

### rpS6 phosphorylation is dispensable for T cell development

**FIGURE 1.**
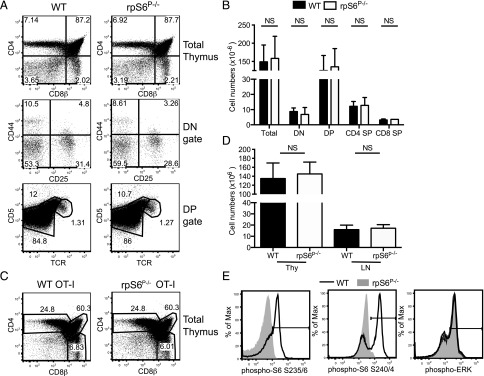
T cell development is independent of rpS6 phosphorylation. FACS analysis of thymocyte populations in WT and rpS6^P−/−^ mice was performed by gating on total live cells or on DN or DP populations as indicated. Representative dot plots showing proportions (**A** and **C**) and quantification of absolute cell numbers (**B** and **D**) of WT and rpS6^P−/−^ polyclonal (A and B) and OT-1 (C and D) thymocyte populations and LN T cells. In bar charts, values represent means and error bars represent SD (*n* > 6 mice/group). *p* > 0.05 as analyzed by Students *t* test. (**E**) Levels of phosphorylation of rpS6 S235/6, S240/4, and ERK T202/Y204 following 30-min stimulation of WT and rpS6^P−/−^ LN OT-1 T cells with 1 μM N4 peptide were assessed by intracellular staining and FACS analysis. Histograms are representative of three replicate experiments.

Expression of rpS6 is essential for T cell development (20), yet the role of inducible phosphorylation of the protein has not been determined. rpS6^P−/−^ knockin mice (21), which have all five Ser residues that can be modified by phosphorylation mutated to Ala, were bred onto the C57BL/6J background for at least eight generations and the impact of rpS6 phosphorylation on T cell development assessed. FACS analysis demonstrated similar proportions and numbers of CD8^−^CD4^−^ double-negative (DN), CD8^+^CD4^+^ double-positive (DP), and CD8^+^CD4^−^ and CD8^−^CD4^+^ single-positive (SP) thymocytes in WT and rpS6^P−/−^ knockin thymi (Fig. 1A, 1B). Furthermore, the distribution of DN1–4 populations, as discriminated by expression of CD44 and CD25, was not altered in rpS6^P−/−^ knockin mice (Fig. 1A). DP thymocytes can be subdivided into DP1–3 populations by their surface expression of TCR and CD5 (31). As with other thymocyte subpopulations, progression from DP1–3 was independent of rpS6 phosphorylation as evident by the presence of similar proportions of these populations in WT and rpS6^P−/−^ thymi suggesting that positive selection was proceeding normally (Fig. 1A).

To confirm that rpS6 phosphorylation was not required for positive selection, we crossed rpS6^P−/−^ knockin mice to an OT-1 *Rag1^−/−^* TCR transgenic background. As with mice with a polyclonal TCR repertoire, WT and rpS6^P−/−^ OT-1 mice had similar distributions and numbers of thymocyte populations (Fig. 1C, 1D). To check that the knockin mice were unable to phosphorylate rpS6, we stimulated OT-1 LN cells with cognate SIINFEKL peptide that induced robust phosphorylation of rpS6 in WT but not rpS6^P−/−^ OT-1 T cells as assessed using two distinct phospho-specific mAbs (Fig. 1E). As a control, we showed that levels of TCR-induced phospho-ERK were similar in WT and rpS6^P−/−^ cells (Fig. 1E). Taken together, these data indicate that rpS6 phosphorylation is dispensable for thymic T cell development.

**FIGURE 2.**
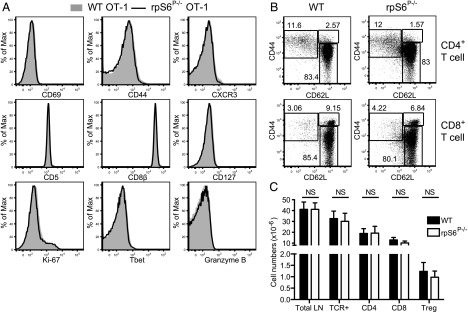
Comparable phenotype of WT and rpS6^P−/−^ peripheral T cells. (**A**) Histograms show levels of expression of surface markers CD69, CD44, CXCR3, CD5, CD8β, CD127, and intracellular expression of Ki-67, Tbet, and granzyme B by gated LN OT-1 T cells. (**B**) Dot plots show distribution of naive and memory populations on gated CD4^+^ and CD8^+^ polyclonal T cells from WT and rpS6^P−/−^ mice. (**C**) Quantification of polyclonal LN T cell populations in WT and rpS6^P−/−^ mice. Values represent mean and error bars SD (*n* = 6). *p* > 0.05 as determined using Student *t* test.

Consistent with the analysis of T cell development, mature T cells were present in similar numbers in the LNs of WT and rpS6^P−/−^ OT-1 mice (Fig. 1D). Both WT and rpS6^P−/−^ LN OT-1 T cells were phenotypically naive expressing low levels of activation markers CD44, CD69, CXCR3, effector protein granzyme B, and transcription factor Tbet and similar levels of CD8, CD127, and CD5 (Fig. 2A). Moreover, the proportions of LN polyclonal naive (CD44^low^CD62L^+^), central memory (CD44^high^CD62L^+^), and effector memory (CD44^high^CD62L^−^) CD4^+^ and CD8^+^ T cells (Fig. 2B) and numbers of regulatory T cells (Fig. 2C) were indistinguishable in C57BL/6 WT and rpS6^P−/−^ mice. Furthermore, surface expression of TCR, coreceptors, and CD5 and cell size, as assessed by forward scatter, of resting WT and rpS6^P−/−^ polyclonal CD4^+^ and CD8^+^ T cells were similar (Supplemental Fig. 1). Finally, the proportions of T cells expressing the proliferation-associated Ag Ki-67 were similar in WT and rpS6^P−/−^ mice (Fig. 2A, Supplemental Fig. 1), indicating that rpS6 phosphorylation does not influence basal T cell number, turnover, or phenotype.

### rpS6 phosphorylation is not required for TCR-induced responses in vitro

**FIGURE 3.**
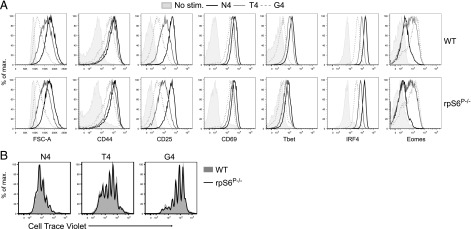
rpS6 phosphorylation is dispensable for T cell activation in response to strong, intermediate, and weak TCR agonist peptides. LN OT-1 T cells were stimulated with 1 μM N4, T4, or G4 for 24 h (**A**) or 72 h (**B**) prior to FACS analysis. Histograms show FSC and levels of surface expression of activation markers (CD44, CD25, and CD69) or intracellular expression of transcription factors (Tbet, IRF4, and Eomesodermin [eomes]) on gated live CD8^+^ T cells (A). For analysis of cell proliferation, dilution of CellTrace Violet was assessed (B). Data are representative of one of four repeated experiments.

Previous data have shown that rpS6 phosphorylation regulates cell size in fibroblasts (21), whereas rapamycin treatment reduces cell size under conditions of T cell activation. Furthermore, T cell blasting after TCR triggering imposes huge metabolic demands that are regulated by mTOR-dependent signaling pathways (1). Using peptides of varying affinity for the OT-1 TCR, recent work showed that TCR signaling strength determines the extent of upregulation of key transcription factors such as IFN regulatory factor (IRF)4 and the gain of effector cell function in CD8^+^ T cells via mTOR (32). We sought to assess the role of rpS6 phosphorylation in these processes using three variants of ova-peptide: high-affinity SIINFEKL (N4), intermediate-affinity SIITFEKL (T4), and very low affinity SIIGFEKL (G4). Following 24 h of activation in vitro with N4, T4, or G4 peptides, the extent of WT OT-1 T cell activation was assessed by FACS analysis of activation marker and transcription factor expression. As expected, in WT cells, cell size as assessed by the forward scatter (FSC) parameter, the levels of surface CD25, CD69, CD44, and intracellular expression of key transcription factors Tbet and IRF4 were greatest following N4 stimulation and lowest following G4 stimulation (Fig. 3A). By contrast and as reported previously (33), the levels of eomesodermin expression following stimulation were inversely correlated with Ag affinity (Fig. 3A). Following N4 stimulation, Tbet levels were very modestly but significantly decreased (on average ∼16% as calculated using mean fluorescence intensity, *p* < 0.01 from *n* = 4 experiments) in rpS6^P−/−^ as compared with WT OT-1 T cells (Fig. 3A). However, no such differences were apparent following either T4 or G4 stimulation. Furthermore, cell size and the levels of activation marker and additional transcription factor expression were comparable in WT and rpS6^P−/−^ OT-1 T cells under all conditions. Further analyses demonstrated that the extent of peptide-induced proliferation, as assessed by FACS analysis of CellTrace Violet dilution following 72 h of stimulation, was also very similar in WT and rpS6^P−/−^ T cells (Fig. 3B).

**FIGURE 4.**
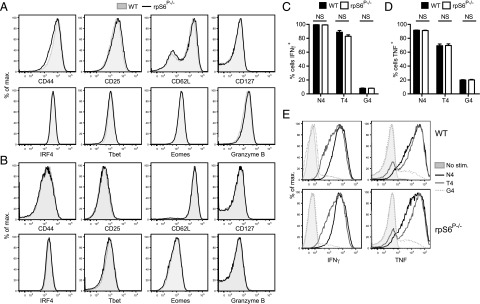
Differentiation of effector CTLs does not require rpS6 phosphorylation. LN OT-1 T cells were stimulated for 2 d with N4 peptide, followed by differentiation for 4 d in IL-2 (**A**) or IL-15 (**B**). FACS analysis shows levels of expression of surface activation markers and intracellular transcription factors and effector proteins by gated live CTLs. IL-2–generated CTLs were restimulated for 4 h with 1 μM N4, T4, or G4 peptides and levels of intracellular IFN-γ and TNF assessed by FACS (**C**–**E**). In bar charts, values representative means and error bars SD (*n* = 3) from one of five replicate experiments. *p* > 0.05 as determined by Student *t* test.

As mTOR is known to regulate effector-memory cell fate decisions, we generated CTLs in vitro by stimulating OT-1 T cells with N4 peptide for 2d, followed by an additional 4-d culture in high doses of either IL-2 or IL-15. Activation of CD8^+^ T cells in the presence of high doses of IL-2 promotes differentiation to effector and effector-memory–like phenotypes, whereas high dose of IL-15 induce a central-memory–like phenotype in vitro (34, 35). Under these conditions, WT and rpS6^P−/−^ cell populations expanded to a similar extent (data not shown). Differentiation in IL-2, as compared with IL-15, resulted in higher expression of CD44, CD25, granzyme B, Tbet, IRF4, and eomesodermin and lower levels of L-selectin (CD62L) by CTL (Fig. 4A, 4B). Under both conditions, the phenotype of WT and rpS6^P−/−^ effector CTLs was indistinguishable (Fig. 4A, 4B). Furthermore, the proportions of IFNγ^+^ and TNF^+^ CTLs (Fig. 4B) and levels of cytokine production per cell (Fig. 4C) upon N4, T4, and G4 restimulation were similar for WT and rpS6^P−/−^ populations. These results indicate that rpS6 phosphorylation is not required for T cell growth, activation, differentiation, and effector function in response to peptides of high, intermediate, and weak affinity.

### Polyclonal T cell responses to infection do not require rpS6 phosphorylation

**FIGURE 5.**
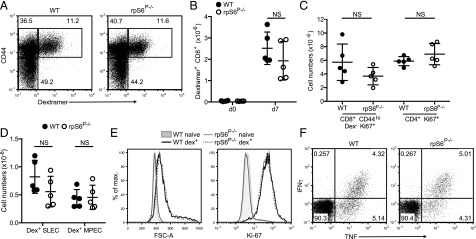
T cell activation in *Listeria* infection is independent of rpS6 phosphorylation. Groups of WT and rpS6^P−/−^ mice were infected i.v. with LmOva and ova-specific T cell responses analyzed at day 7 postinfection. (**A**) Dot plots show representative analysis of the proportions of naive CD44^low^, effector CD44^high^, and ova-specific CD44^high^ H-2K^b^-ova dextramer^+^ population on gated CD8^+^ spleen T cells from infected mice. Absolute quantification of spleen dextramer (dex)^+^CD8^+^ T cells (**B**), CD8^+^ dex^−^CD44^hi^ Ki67^+^, and CD4^+^ Ki67^+^ (**C**) in WT and rpS6^P−/−^ mice. (**D**) Quantification of dex^+^ CD127^−^KLRG1^+^ short-lived effector cells (SLEC) and CD127^+^KLRG1^−^ memory progenitor effector cells (MPEC). Circles represent values from individual mice and lines the mean of each genotype (*n* = 5/group), *p* > 0.05, as determined by Student *t* test. Histograms show cells size (FSC-A) and levels of intracellular Ki-67 on gated naive and dex^+^CD8^+^ splenic T cells from day 7 infected mice (**E**). Splenocytes were restimulated with 1 μM N4 peptide; dot plots show levels of intracellular IFN-γ and TNF by gated CD8^+^ T cells (**F**). All data are from one of two replicate experiments.

In vitro results could be masked by metabolic supersufficiency of culture conditions. To determine the role of rpS6 phosphorylation in T cell immune responses in vivo, we infected WT C57BL/6J and rpS6^P−/−^ mice with an ova-expressing strain of *L. monocytogenes*. Primary splenic CD8^+^ T cell responses to the immunodominant SIINFEKL peptide were monitored using MHC class I–peptide dextramers. Numbers and proportions of dextramer-positive effector CD8^+^ T cells were comparable in WT and rpS6^P−/−^ mice as measured at 7 d postinfection (Fig. 5A, 5B). Furthermore, numbers of actively proliferating (Ki-67^+^) CD8^+^CD44^hi^ dextramer-negative and CD4^+^ cells were comparable in WT and rpS6^P−/−^ mice, suggesting that the overall T cell response to Lm-Ova infection was similar in both groups (Fig. 4C). Importantly, numbers of KLRG^+^CD127^−^ short-lived effector cells and KLRG1^-^CD127^+^ memory progenitor effector cells within the dextramer-positive CD8^+^ T cell population were similar in WT and rpS6^P−/−^ mice (Fig. 5D). The cell size (FSC) and levels of expression of Ki-67^+^ in WT and rpS6^P−/−^ ova-specific effector CD8^+^ T cell were also indistinguishable (Fig. 5E). Finally, levels of IFN-γ and TNF production following in vitro recall responses to SIINFEKL were also not affected by the rpS6^P−/−^ mutations (Fig. 5F). These data indicate that rpS6 phosphorylation is not required for the generation of effective polyclonal T cell responses to in vivo infection.

### rpS6^P−/−^ T cells are fully sensitive to mTORC1 and S6K1 inhibition

**FIGURE 6.**
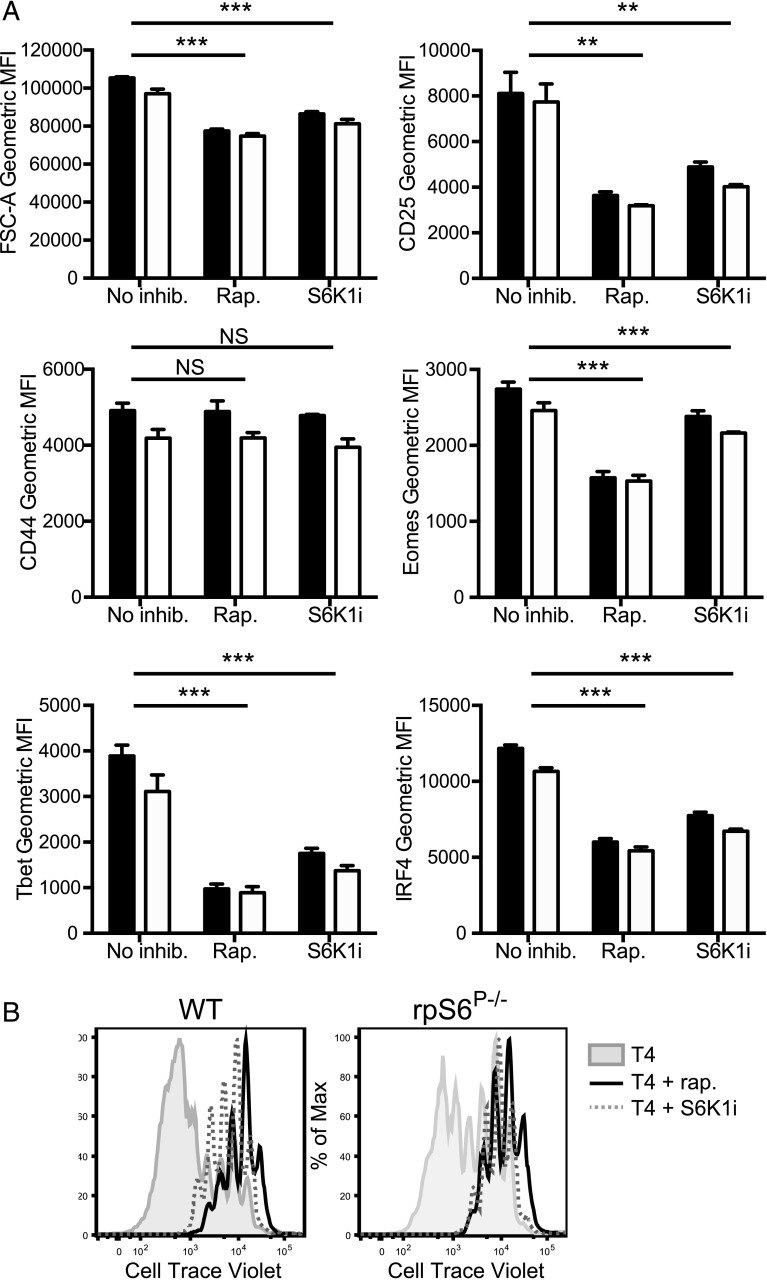
WT and rpS6^P−/−^ OT-1 T cells are equally sensitive to the effects of mTOR and S6K1 inhibition. LN OT-1 T cells were stimulated with 1 μM T4 peptide in the presence or absence of rapamycin (rap) or S6K1 inhibitor (S6K1i) for 24 h (**A**) or 72 h (**B**). Levels of expression of activation markers and transcription factors are represented as geometric mean fluorescence intensity (MFI) as assessed by FACS (A). In bar charts, values represent means and error bars SD (*n* = 3). **p < 0.01, ***p < 0.001 as determined by Student *t* test. In each case, statistical analyses represent comparison of no inhibitor versus rapamycin or S6K1i-treated cells for both WT and rpS6^P−/−^ cells. Proliferation was assessed by dilution of CellTrace Violet (B). All data are from one of three repeated experiments.

The data suggested that mTORC1 function in T cells was independent of the ability of this pathway to induce rpS6 phosphorylation. It was possible that mTORC1 could regulate T cell activation via S6K1-independent pathways (e.g., via regulation of 4E-binding proteins) or via S6K1-dependent rpS6 phosphorylation–independent mechanisms. For example, S6K1 can modulate gene expression via the phosphorylation of additional substrates such as elongation factor 2 kinase (22). To discriminate between S6K1-dependent and -independent effects of mTORC1, we compared the effects of rapamycin and a highly-specific S6K1 inhibitor (28) on WT and rpS6^P−/−^ OT-1 T cell responses. Treatment of OT-1 T cells with rapamycin reduced TCR-driven cell growth as assessed by FSC and the upregulation of CD25, Tbet, IRF4 and eomesodermin but had no impact upon levels of CD44 (Fig. 6A). As expected, rapamycin also slowed the rate of T cell proliferation (Fig. 6B). Treatment with the S6K1 inhibitor PF-4708671 also inhibited these parameters albeit to a somewhat reduced extent. Importantly, the extent of inhibition of WT and rpS6^P−/−^ cells by either rapamycin or PF-4708671 was similar. Therefore, mTORC1 and S6K1 are important regulators of T cell activation and differentiation whereas rpS6 phosphorylation is dispensable for these processes.

## Discussion

mTOR signaling pathways are central to the regulation of T cell growth, metabolism, differentiation, and memory. An understanding of the effector mechanisms that regulate these processes is essential for the development of more sophisticated and targeted therapies to manipulate mTOR activation and T cell responses in the clinic. We sought to determine the role of downstream effectors of mTORC1 and, surprisingly, found that phosphorylation of the canonical target of the S6K1 signaling axis, rpS6, was dispensable for T cell development, activation, differentiation, and effector function both in vitro and in vivo.

More than 20 y ago, it was hypothesized that rpS6 phosphorylation is an important regulator of the translation of mRNAs containing a 5′-terminal oligopyrimidine tract (36). However, analysis of rpS6^P−/−^ mice demonstrated that rpS6 phosphorylation is dispensable for the translation of 5′-terminal oligopyrimidine tract mRNAs and plays only a minor regulatory role in global protein synthesis (21). Nonetheless, these studies showed that rpS6 phosphorylation regulates the cell size of several cell types including pancreatic β cells, murine embryonic fibroblasts (MEFs) and hepatocytes (21, 22, 37). Thus, rpS6^P−/−^ MEFs were smaller than WT counterparts and their size was not further reduced by rapamycin (21), indicating that mTORC1-dependent regulation of MEF cell size is entirely mediated via rpS6 phosphorylation. By contrast, our data show that rpS6^P−/−^ and WT T cells are similar in cell size both under basal conditions and following Ag-induced stimulation. Furthermore, whereas rpS6^P−/−^ MEFs have an elevated rate of cell cycle progression and proliferation compared with WT MEFs, rpS6^P−/−^ and WT T cells have comparable proliferative responses to TCR stimulation and mitogenic cytokines such as IL-2. Indeed, we found that, in all parameters of TCR-induced activation, differentiation, and effector function assessed in the current work, rpS6^P−/−^ and WT T cell responses were comparable.

These results raise the question of what are the important downstream effectors of mTORC1 if rpS6 phosphorylation is dispensable for T cell activation? Araki et al. (13) reported that small interfering RNA–induced knockdown of S6K1 enhanced CD8^+^ T cell memory differentiation in a similar manner to rapamycin treatment. Furthermore, expression of a constitutively active form of S6K1 in CD4^+^ T cells counteracted the inhibitory effects of rapamycin on Th17 differentiation (38). In the current work, our data using a specific pharmacological inhibitor indicate that S6K1 is important for Ag-induced OT-1 T cell activation and proliferation. Besides rpS6, S6K1 has several additional downstream substrates including elongation factor 2 kinase (39), initiation factor eIF4B (40), and programmed cell death 4 (PDCD4) (41) that regulate translation rates. Indeed, recent data have shown that mTORC1-mediated regulation of both 4E-BP function and phosphorylation of eIF4B and PDCD4 are required to sustain global rates of protein synthesis (42). Furthermore, S6K1 modulation of eIF4B phosphorylation regulates the expression of the key transcription factor cMyc in cancer cells (43). Interestingly, micro-RNA 21 suppresses the expression of the S6K1 substrate and translational inhibitor PDCD4 to regulate cell survival in T cell leukemia (44), whereas elevated levels of T cell micro-RNA 21 expression and subsequent suppression of PDCD4 are associated with active disease in systemic lupus erythematosus (45). Thus, the combination of multiple downstream targets is likely to be essential for the effects of the mTORC1/S6K1 signaling axis in T cells. Furthermore, mTORC1-induced 4E-BP1 phosphorylation is likely to be important in the regulation of immune responses. Indeed, innate production of type I IFNs is translationally repressed by 4E-BPs, whereas in the combined absence of 4E-BP1 and 4E-BP2, levels of IFN-α and -β are enhanced and viral replication suppressed (46). Interestingly, rapamycin is a poor inhibitor of 4E-BP1 phosphorylation (47), suggesting that the effects of this drug may primarily be mediated via effects on the S6K1 axis.

In conclusion, our data show that mTORC1 and S6K1 influence T cell activation and differentiation independently of their capacity to mediate rpS6 phosphorylation. Further analysis of the role of additional downstream effectors of this pathway in T cells may provide insight into the targets of this pathway that are important for T cell function.

## Supplementary Material

Data Supplement
