# Supplementary material for: Mechanistic Target of Rapamycin Complex 1/S6 Kinase 1 Signals Influence T Cell Activation Independently of Ribosomal Protein S6 Phosphorylation
Source: J Immunol. 2015 Oct 9;195(10):4615–22. doi: 10.4049/jimmunol.1501473 (PMC4635570; doi:10.4049/jimmunol.1501473)
Supplement: Data Supplement [file JI_1501473.zip › JI_1501473_Supplemental_Figure_1.pdf]

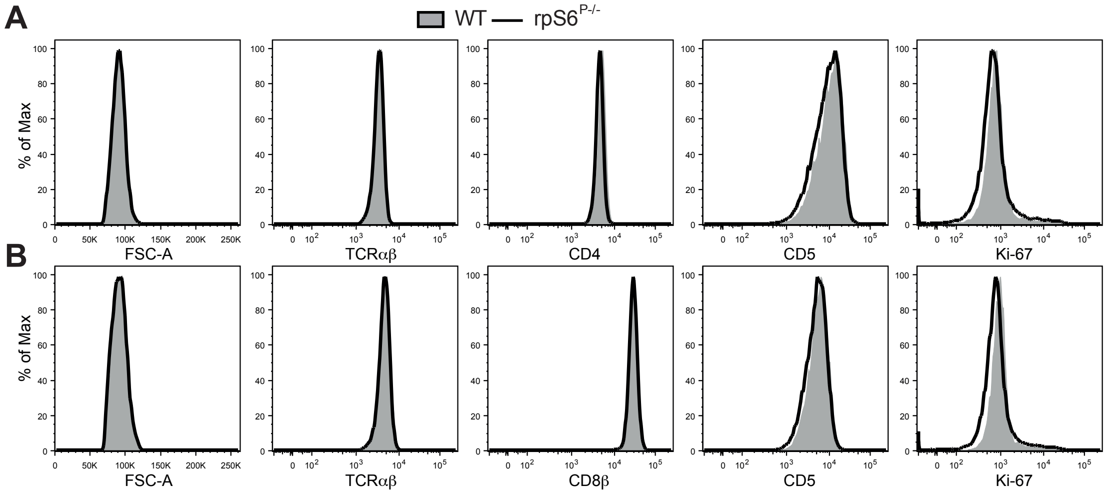


**Supplementary Figure 1:** rpS6 phosphorylation does not impact upon the basal phenotype of polyclonal $CD4^+$ and $CD8^+$ T cells. FACS histograms show cell size (FSC-A), surface expression of TCR, CD4, CD8β and CD5, and intracellular expression of Ki-67 by gated $CD4^+$(*A*) and $CD8^+$(*B*) lymph node T cells from 7 week old mice.
